# Impact of a reminder/extinction procedure on threat-conditioned pupil size and skin conductance responses

**DOI:** 10.1101/lm.050211.119

**Published:** 2020-04

**Authors:** Josua Zimmermann, Dominik R. Bach

**Affiliations:** 1 Computational Psychiatry Research, Department of Psychiatry, Psychotherapy, and Psychosomatics, Psychiatric Hospital, University of Zurich, 8032 Zurich, Switzerland; 2 Neuroscience Centre Zurich, University of Zurich, 8057 Zurich, Switzerland; 3 Wellcome Centre for Human Neuroimaging and Max Planck/UCL Centre for Computational Psychiatry and Ageing Research, University College London, London WC1 3BG, United Kingdom

## Abstract

A reminder can render consolidated memory labile and susceptible to amnesic agents during a reconsolidation window. For the case of threat memory (also termed fear memory), it has been suggested that extinction training during this reconsolidation window has the same disruptive impact. This procedure could provide a powerful therapeutic principle for treatment of unwanted aversive memories. However, human research yielded contradictory results. Notably, all published positive replications quantified threat memory by conditioned skin conductance responses (SCR). Yet, other studies measuring SCR and/or fear-potentiated startle failed to observe an effect of a reminder/extinction procedure on the return of fear. Here we sought to shed light on this discrepancy by using a different autonomic response, namely, conditioned pupil dilation, in addition to SCR, in a replication of the original human study. *N* = 71 humans underwent a 3-d threat conditioning, reminder/extinction, and reinstatement, procedure with 2 CS+, of which one was reminded. Participants successfully learned the threat association on day 1, extinguished conditioned responding on day 2, and showed reinstatement on day 3. However, there was no difference in conditioned responding between the reminded and the nonreminded CS, neither in pupil size nor SCR. Thus, we found no evidence that a reminder trial before extinction prevents the return of threat-conditioned responding.

According to a dynamic memory view, consolidated memory traces are malleable, rendered labile by retrieval or reactivation (Nader 2015). Under physiological conditions, this is spontaneously followed by a new phase of stabilization, termed reconsolidation. During this time window, local protein synthesis inhibition with anisomycin can interfere with, or erase, memory (Nader et al. 2000; Nader 2015). This has been particularly demonstrated for aversive Pavlovian conditioning, also termed fear conditioning, which provides a cross-species model of amygdala-dependent memory (Delgado et al. 2006) with translational relevance for psychiatric conditions such as posttraumatic stress disorder (Foa et al. 1989). Reconsolidation blockade thus could provide a potentially powerful way of treating clinical conditions that involve maladaptive aversive memory. This has motivated a search for drugs (Dębiec and Ledoux 2004; Brunet et al. 2008; Kindt et al. 2009) or behavioral interference manipulations (Monfils et al. 2009; Schiller et al. 2010; Schiller and Phelps 2011) that are applicable in humans and can disrupt threat memory during the reconsolidation window. One of these behavioral procedures is a nonreinforced presentation of the conditioned stimulus (CS+) to initiate the reconsolidation process, followed 10 min later by a standard extinction protocol (nonreinforced CS+ and CS− presentations). Without a preceding reminder cue, behavioral expression of conditioned responses vanishes during extinction, but the underlying fear memory regularly persists and reappears after reinstatement of the unconditioned stimulus (US), or after passage of time (recovery) (Myers and Davis 2007). In contrast, both in rats (Monfils et al. 2009) and humans (Schiller et al. 2010), a reminder cue 10 min before extinction prevented conditioned responding to reappear over time (no spontaneous recovery) or after reinstatement. In the human study, this lack of reinstatement was stable over a year and was specific to the reminded cue (Schiller et al. 2010). This may suggest that extinction training during the reconsolidation period overwrites the original fear memory, in contrast to extinction training outside this time window.

While this appears a potentially promising route to manipulate maladaptive memory in clinical settings, the generalizability of initial findings in humans remains unclear (Lee et al. 2017). Several studies directly or conceptually replicated the impact of a preextinction reminder cue on the persistence of fear memory (Agren et al. 2012a,b; Oyarzún et al. 2012; Schiller et al. 2013; Steinfurth et al. 2014; Johnson et al. 2015). However, a comparably large number of similarly powered studies failed to replicate this finding (Soeter and Kindt 2011; Golkar et al. 2012; Kindt and Soeter 2013; Meir Drexler et al. 2014; Klucken et al. 2016; Kroes et al. 2017). Post hoc, this discrepancy has been explained with several possible differences in the experimental protocol (Lee et al. 2017). A meta-analysis revealed a significant effect of reminder/extinction compared with standard extinction procedure, moderated by several other variables, among them the type of CS (Kredlow et al. 2016): none of the studies using “fear-relevant” CS, i.e., pictures of spiders or snakes, replicated the reminder/extinction effect on fear memory. Another factor, not considered in this meta-analysis, is that all studies replicating the reminder/extinction effect used skin conductance responses (SCR) to assess conditioned responding, while none of the several published experiments using fear-potentiated startle replicated the finding. Here, we focused on this latter discrepancy.

Crucially, threat-conditioned defensive responses in humans can be assessed in a number of different ways, among which SCR (Bach et al. 2010; Boucsein 2012) and fear-potentiated startle (Brown et al. 1951; Bach 2015; Khemka et al. 2017) are used most frequently. Other measures include pupil size response (PSR) (Korn et al. 2017), phasic bradycardia (Castegnetti et al. 2016) and respiration amplitude responses (Castegnetti et al. 2017). While all of these measures differ between CS+ and CS− in standard fear conditioning and retention, trial-by-trial trajectories suggest they may relate to different underlying components of the learning process (Li et al. 2011; Zhang et al. 2016; Bach et al. 2018b; Leuchs et al. 2019, for review, see Ojala and Bach 2019). Also, they appear to be differentially affected by pharmacological interventions targeting consolidation (Bach et al. 2018b) and reconsolidation (Soeter and Kindt 2010). Furthermore, a direct comparison has revealed that PSR may have higher accuracy in inferring fear memory than SCR (Korn et al. 2017). This is why we here sought to replicate the findings of Schiller et al. (2010) with PSR as a measure of fear memory. We used a similar experimental setup to their within-subjects experiment 2 but optimized some parameters in light of a recent meta-analysis by Kredlow et al. (2016), in order to increase the chances of revealing success of the reminder/extinction procedure (see Table 1). Specifically, we used a longer US duration and an increased number of acquisition trials. Furthermore, Schiller et al. (2010) reminded both the CS− and the CSr+ in their second experiment. In line with previous replication studies, inluding a follow-up study by Schiller et al. (2013), we only reminded CSr+. Additionally, we shortened the intertrial interval (ITI) compared to Schiller et al. (2010), as our approach of using a general linear model (GLM) instead of peak scoring to quantify conditioned responses enables us to handle shorter ITIs. Our sample size was based on the signal-to-noise ratio of PSR under control conditions. Thus, we recruited a sample that provided 85% power to detect an at least 50% absolute reduction of fear retention, corresponding to the ∼60% reduction found in Schiller et al. (2010).

**Table 1.**
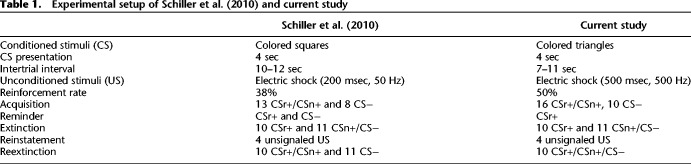
Experimental setup of Schiller et al. (2010) and current study

## Results

**Figure 1.**
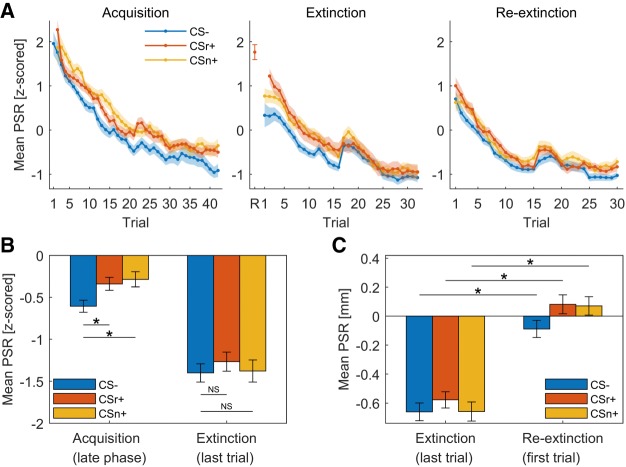
Pupil size responses averaged over all participants. Error shades/bars indicate standard error of the mean. All analyses within session are based on *z*-scored data to enhance sensitivity; all analyses across session are based on untransformed data. Only unreinforced CS+ trials were analyzed. (*A*) Grand mean PSR per condition for each trial (*z*-scored within session). Missing data points were imputed for plotting, using previous neighbor interpolation. The first trial of extinction (R) for CSr+ reflects the retrieval trial. Note that in acquisition, there is no data for the first CS+ trials as this was always reinforced. (*B*) Grand mean PSR averaged over the late phase of acquisition (second half) and last trial of extinction (*z*-scored within session). Participants showed successful fear acquisition. Both CS+ conditions evoked significantly higher responses than CS−. During the last trial of extinction, PSR to both CS+ were similar to CS−, indicating extinction. (*C*) Fear recovery index according to Schiller et al. (2010) using nonnormalized data: difference between the last trial of extinction and the first trial of reextinction after reinstatement. Positive values: pupil dilation; negative values: pupil constriction. Fear responses recovered equally for all three conditions after reinstatement. Reminded (CSr+) and nonreminded (CSn+) stimuli did not differ with respect to fear recovery.

**Figure 2.**
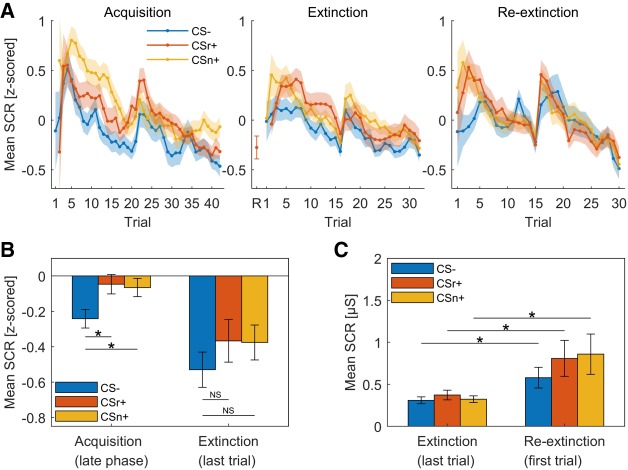
Skin conductance responses averaged over all participants. Error shades/bars indicate standard error of the mean. All analyses within session are based on *z*-scored response estimates to enhance sensitivity; all analyses across session are based on untransformed estimates. Only unreinforced CS+ trials were analyzed. (*A*) Grand mean SCR per condition for each trial (*z*-scored within session). Missing data points were imputed for plotting, using previous neighbor interpolation. The first trial of extinction (R) for CSr+ reflects the retrieval trial. Note that in acquisition, there is no data for the first CS+ trials as this was always reinforced. (*B*) Grand mean SCR averaged over the late phase of acquisition (second half) and last trial of extinction (*z*-scored within session). Participants showed successful fear learning. The responses to both CS+ conditions were significantly higher than CS− during the second half of acquisition and diminished to a similar level compared to CS− in the last trial of extinction. (*C*) Fear recovery index according to Schiller et al. (2010): difference of grand mean responses between the last trial of extinction and the first trial of reextinction after reinstatement. Nonnormalized response estimates were used for the 2-d comparison. Fear responses recovered significantly for all three conditions after reinstatement and comparing the recovery of fear for the reminded (CSr+) and the nonreminded (CSn+) stimuli did not reveal any difference.

Participants were trained on two CS+ (CSr+/CSn+) and one CS− on day 1. On subsequent day 2, CSr+ was reminded and 10 min later, extinction training started with all 3 CS and no reinforcement. After reinstatement on subsequent day 3, we measured fear recovery under extinction (termed here reextinction). For all sessions, we did not instruct participants about the CS identities, or about the number of reinforced CS. Results for PSR are shown in Figure 1 and Table 2; results for SCR in Figure 2 and Table 3.

**Table 2.**
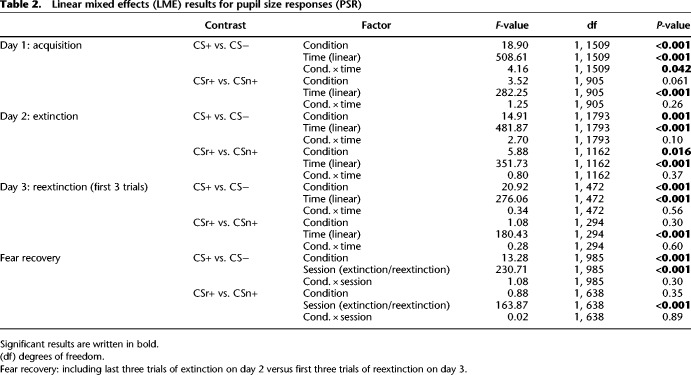
Linear mixed effects (LME) results for pupil size responses (PSR)

**Table 3.**
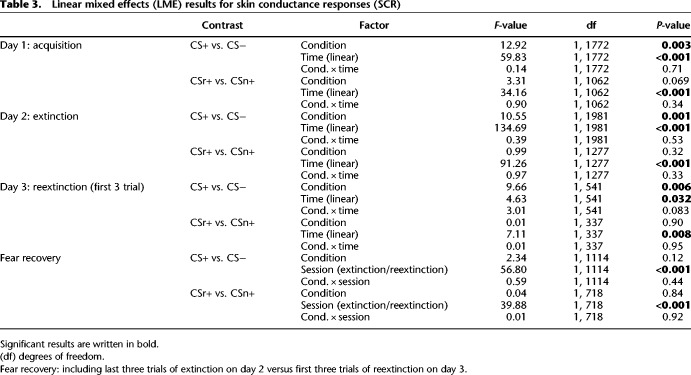
Linear mixed effects (LME) results for skin conductance responses (SCR)

### Fear acquisition (day 1)

PSR on day 1 showed successful fear learning with significantly larger phasic pupil dilation for CS+ versus CS− and a condition × time interaction (see Table 2). Unexpectedly, CSn+ evoked almost significantly stronger responses than CSr+ (see Table 2), even though both stimuli were colored triangles and colors were counterbalanced across subject. Follow-up analyses indicated this difference was due to responses in the first trial of each condition, and it disappeared when excluding the first trial, or when analyzing the second half of acquisition alone (second half of acquisition: CSr+ vs. CS−: *t*_(69)_ = 3.13, *P* = 0.003; CSn+ vs. CS−: *t*_(69)_ = 2.91, *P* = 0.005; CSr+ vs. CSn+: *t*_(69)_ = 0.54, *P* = 0.59) (see Supplemental Table S1 for the results of a linear mixed-effects model (LME)). Similarly, SCR were higher for CS+ than CS−, and almost significantly larger for the CSn+ than CSr+ (see Table 3). Again, this difference was due to early acquisition trials and was absent when excluding the first trial or the second half of acquisition trials (second half acquisition: CSr+ vs. CS−: *t*_(70)_ = 2.25, *P* = 0.027; CSn+ vs. CS: *t*_(70)_ = 2.00, *P* = 0.049; CSr+ vs. CSn+: *t*_(70)_ = 0.29, *P* = 0.78) (see Supplemental Table S2 for LME results). Thus, we conclude that the two CS+ were ultimately learned to the same extent.

### Fear extinction after retrieval (day 2)

CSr+ was reminded 10 min before extinction. During extinction, we observed higher PSR for CS+ than CS−, and an overall decrease over time (main effects CS+/− and time). Nonreminded CSn+ evoked higher PSR during extinction than CSr+, but a follow-up *t*-test revealed no difference when comparing the last extinction trial for both conditions (*t*_(64)_ = 0.97, *P* = 0.34). In the last extinction trial, PSR to either CS+ were not significantly different from CS− (CSr+ vs. CS−: *t*_(64)_ = 1.45, *P* = 0.15; CSn+ vs. CS−: *t*_(64)_ = 0.24, *P* = 0.81).

SCR were overall higher to CS+ than CS− (main effect CS+/−) and declined over time for all conditions (main effect time). There was no overall difference between CSr+ and CSn+. In the last extinction trial, SCR to CSr+ and CSn+ did not differ (*t*_(63)_ = 0.06, *P* = 0.95), and SCR to both CS+ were not significantly different from CS− (CSr+ vs. CS−: *t*_(63)_ = 1.02, *P* = 0.31; CSn+ vs. CS−: *t*_(63)_ = 1.20, *P* = 0.23).

We conclude that threat memory was retained on day 2 (overall CS+/CS− difference), while during extinction, responses to all CS decreased to a comparable magnitude.

### Fear recovery (day 3)

The main outcome of our experiment was the fear recovery test. First, we tested if responses to reminded (CSr+) and nonreminded (CSn+) stimuli diverged in the first three trials of the reextinction session after reinstatement on day 3. Next, we analyzed fear recovery by combining the last three trials of extinction for each condition, and the first three trials of reextinction, into a linear mixed effect model. Finally, we repeated the analysis performed in Schiller et al. (2010): a two-way ANOVA with main effects condition (CS−, CSr+, CSn+) and time (first and second half) for the reextinction session, followed by one-sample *t*-tests of the fear recovery index (last trial extinction minus first trial reextinction, separately for each condition). The results of the remaining part of the reextinction session are reported in Supplemental Tables S3 and S4.

During the first three trials after reinstatement, PSR were higher for CS+ compared to CS− (see Table 2), and both CS+ evoked significantly higher responses than CS− (CSr+: *F*_(2,287)_ = 21.60, *P* < 0.001; CSn+: *F*_(2,294)_ = 12.08, *P* < 0.001), but there was no difference between CSr+ and CSn+ (see Table 2). Comparing model evidence for the full model with a reduced model that did not separate CSr+ and CSn+, evidence was in favor of the reduced model (log Bayes factor (LBF) = 5.31). Fear recovery analysis revealed a strong difference between CS+ versus CS− and significantly higher responses after reinstatement for all CS, but no condition × session interaction (see Table 2). CSr+ and CSn+ did not differ in fear recovery analysis, even at trend-level (see Table 2), and model evidence favored a reduced model (LBF = 5.90). In an analysis equivalent to Schiller et al. (2010), we found a significant main effect of condition (*F*_(2,390)_ = 3.76, *P* = 0.02) and time (*F*_(1,390)_ = 72.83, *P* < 0.001), but not the condition × time interaction observed in Schiller et al. (2010). In *t*-tests of the fear recovery index, we observed significantly stronger responses after reinstatement for all three conditions (CS−: *t*_(61)_ = 6.95, *P* < 0.001; CSr+: *t*_(61)_ = 7.47, *P* < 0.001; CSn+: *t*_(61)_ = 7.91, *P* < 0.001). When testing CSr+ against CSn+ in the difference between the last trial of extinction and the first trial after reinstatement, we detected no difference in the recovery of fear (*t*_(61)_ = 0.89, *P* = 0.38). In summary, the reminded (CSr+) and the nonreminded stimuli (CSn+) evoked similar responses after reinstatement and did not differ when comparing the increase from extinction to reextinction after reinstatement. The strong difference between CS+ and CS− after reinstatement confirms a reinstatement effect. However, the lack of a condition × session interaction in fear recovery analysis and the significant increase of CS− after reinstatement compared to the end of extinction indicates an additional generalized nondifferential reinstatement effect, where both CS+ and CS− are enhanced.

SCR analysis of the first three trials showed a main effect of CS+/CS− (see Table 3), and both CS+ differed from CS− (CSr+: *F*_(2,337)_ = 5.40, *P* = 0.021; CSn+: *F*_(2,337)_ = 6.55, *P* = 0.011) while there was no difference between CSr+ versus CSn+ (see Table 3). Fear recovery analysis showed significantly higher responses after reinstatement for all CS, but no condition × session interaction, and in contrast to PSR, no main effect of CS+/CS−. Model evidence favored a reduced model for the first three trials of reextinction (LBF = 6.48) and fear recovery (LBF = 5.98). In an analysis equivalent to Schiller et al. (2010), we observed a significant main effect of condition (*F*_(2,402)_ = 3.36, *P* = 0.036) and time (*F*_(1,402)_ = 35.19, *P* < 0.001), but not the condition × time interaction reported in Schiller et al. (2010). All three conditions evoked a significantly higher response after reinstatement compared to the end of extinction (CS−: *t*_(60)_ = 2.23, *P* = 0.029; CSr+: *t*_(60)_ = 2.05, *P* = 0.044; CSn+: *t*_(60)_ = 2.26, *P* = 0.027). Additional comparison between CSr+ and CSn+ revealed no difference (*t*_(60)_ = 1.10, *P* = 0.27).

### Impact of inclusion criteria

Schiller et al. (2010) excluded about 75% of their subjects in experiment 2 by criteria meant to ensure that those analyzed had learned and extinguished the association; finally reporting a sample size of *n* = 18. Since we had not excluded any subjects, we were concerned that our results were not directly comparable to Schiller et al. (2010). Hence, we undertook a supporting analysis after applying the same exclusion criteria as provided in the addendum to their study (Schiller et al. 2018). Based on these criteria, we excluded participants who failed to show successful fear acquisition or extinction. The exclusion criteria and the complete results are listed in the Supplemental material (see Supplemental Table S5 for PSR results and Supplemental Table S6 for SCR results). For the analysis of PSR, we excluded nine participants due to acquisition failure and two participants who did not show adequate extinction. We included only participants with valid data on both days, resulting in 53 participants for days 1 and 2, and 50 participants (due to missing data) on day 3. In line with our main analysis, the first three trials of reextinction and our fear recovery analysis revealed no difference between the reminded and the nonreminded stimuli. In an analysis equivalent to Schiller et al. (2010), we found a significant main effect of condition (*F*_(2,294)_ = 3.73, *P* = 0.03) and time (*F*_(1,294)_ = 33.66, *P* < 0.001), but not the condition × time interaction observed in Schiller et al. (2010). When testing the last trial of extinction to the first trial of reextinction for all three conditions, we observed significantly stronger responses after reinstatement for all three conditions (CS−: *t*_(49)_ = 6.00, *P* < 0.001; CSr+: *t*_(49)_ = 6.21, *P* < 0.001; CSn+: *t*_(49)_ = 6.20, *P* < 0.001). When comparing CSr+ against CSn+ in the difference between the last trial of extinction and the first trial after reinstatement, we detected no difference in the recovery of fear (*t*_(49)_ = 0.20, *P* = 0.84).

For the SCR analysis, we excluded 47 participants due to acquisition failure and two participants because they did not display sufficient extinction, resulting in a final sample of 22 participants. In line with our main analysis, the first three trials of reextinction and our fear recovery analysis revealed no difference between the reminded and the nonreminded stimuli. In an analysis equivalent to Schiller et al. (2010), we observed a significant main effect of time (*F*_(1,126)_ = 18.22, *P* < 0.001) but not of condition and condition × time interaction. We found a significant return of fear for the nonreminded CSn+ stimuli (CSn+: *t*_(21)_ = 2.42, *P* = 0.025), while the reminded stimuli evoked only nonsignificant higher responses after reinstatement (CSr+: *t*_(21)_ = 1.89, *P* = 0.073). CS− responses were similar before and after reinstatement (CS−: *t*_(21)_ = 1.52, *P* = 0.14). However, comparing CSr+ against CSn+ in the difference from the last trial of extinction to the first trial after reinstatement showed equal return of fear for both conditions (CSr+ vs. CSn+: *t*_(21)_ = 0.25, *P* = 0.80).

## Discussion

In this study, we assessed the efficacy of a reminder/extinction procedure to prevent return of fear in humans, by replicating Schiller et al. (2010) with PSR as an additional index of threat conditioning. We recruited a sample size sufficient to detect an at least 50% reduction of fear memory with 85% power and included overall *N* = 66 (PSR) and *N* = 68 (SCR) participants.

Crucially, we found no evidence that a reminder trial before extinction prevents the return of fear in several analyses and both outcome measures. When comparing the last trial of extinction with the first trial after reinstatement for each condition, we observed significant reinstatement equally for the reminded and the nonreminded CS+, but with no difference between the two CS+. Notably, such direct comparison between the two CS+ was not reported in Schiller et al. (2010). Instead, they based their conclusions on post hoc *t*-tests within conditions, after an ANOVA that included all 3 CS and showed a significant CS × early/late reextinction effect. This interaction was not significant in the present study either. To exclude that our negative findings are due to the inclusion of participants that did not successfully learn or extinguish the CS/US associations, we performed a supporting analysis with the same exclusion criteria as Schiller et al. (2010) (published in Schiller et al. (2018)), thus including *N* = 50 participants for PSR and *N* = 22 participants for SCR. This should provide similar or higher power than Schiller et al. (2010) (*N* = 18 participants). After applying these criteria, we did not observe any difference between reminded and nonreminded CS+ either. To summarize, we find no evidence that a reminder/extinction procedure prevents return of fear in our study.

Our findings stand in line with negative replication studies using SCR or fear-potentiated startle in which no effect of reminder/extinction was observed (Soeter and Kindt 2011; Golkar et al. 2012; Kindt and Soeter 2013; Meir Drexler et al. 2014; Klucken et al. 2016; Kroes et al. 2017). Most of these negative studies used fear-relevant CS, highlighted in a recent meta-analysis as an important factor for the inefficacy of the reminder/extinction procedure (Kredlow et al. 2016). Fear-relevant stimuli are learned faster and more resistant to extinction compared to fear-irrelevant stimuli (Lonsdorf et al. 2017). We note that some previous nonreplications used neutral CS (Golkar et al. 2012; Klucken et al. 2016), similar to Schiller et al. (2010) and the present report. Our findings contrast with the SCR results reported in Schiller et al. (2010) and positive replication studies (Agren et al. 2012a,b; Oyarzún et al. 2012; Schiller et al. 2013; Steinfurth et al. 2014; Johnson et al. 2015).

Several reasons may account for this heterogeneity across reminder/extinction studies using SCR, among which is the generally low signal-to-noise ratio of the dependent variable SCR (Staib et al. 2015), which would consequently impact on statistical power (Bach et al. 2018a) and reduce chances of replication (Goodman et al. 2016). As another factor underlying this heterogeneity, small variations in experimental design have been suggested (Lee et al. 2017). A meta-analysis revealed a significant, small-to-moderate effect (*g* = 0.40) of reminder/extinction procedures over standard extinction on the recovery of fear (Kredlow et al. 2016). Besides the impact of fear-relevant CS, this effect was significantly moderated by the number of acquisition trials, US duration, and lack of expectancy ratings. On the other hand, the study design (between/within-subjects), number of extinction trials and duration of reminder trial did not modify the outcome of the reminder/extinction experiments. Regarding the different return of fear tests applied in the studies (reinstatement, spontaneous recovery, reacquisition, renewal), Kredlow et al. (2016) found no evidence that the test type did affect the outcome. In our study, we capitalized on this knowledge to maximize the chances of replicating Schiller et al. (2010): we used a longer US duration (500 msec) and a slightly increased number of acquisition trials (16 trials per CS+ condition) compared to Schiller et al. (2010) (US: 200 msec; 13 trials per CS+ condition).

In Schiller et al. (2010) and subsequent reminder/extinction studies, return of fear was primarily quantified as the difference between the last trial of extinction and the first trial after reinstatement, although psychophysiological measurements are noisy and single-trial comparisons may reduce the robustness of results. On the other hand, the reinstatement effect persists only for a limited time and diminishes quickly over trials, so even only collapsing the first few trials might already underestimate the effect. In our study, we performed both analyses. We first analyzed the last/first three trials without collapsing them to compute the fear recovery index as we wanted to minimize possible distortion of the results due to noise. For direct comparison with previous studies, we included a single-trial comparison. In our study, the single- and multiple-trial comparison yielded the same results, which we believe strengthens the credibility of the conclusions.

Exclusion of participants who do not show adequate fear learning or extinction is common in many laboratories (Schiller et al. 2010; Soeter and Kindt 2011; Oyarzún et al. 2012; Steinfurth et al. 2014). We did not exclude participants in our primary analysis because there is no evidence that participants with no or negative difference between CS+/CS− did not actually learn the association: for example, in our supporting analysis, the same criteria excluded 11 participants in PSR and 49 in SCR, although the underlying learning mechanism is presumably the same. However, we note that it is important to demonstrate successful learning and extinction at least on the group level. Assessment of extinction is however rather heterogeneous across laboratories and studies (Lonsdorf et al. 2017) and the best method for doing so is currently unclear.

As a limitation of our own and previous studies on the topic, we relied on evoking return of fear using reinstatement. In the present experiments, we observed generalized nondifferential return of fear after reinstatement (CS+ and CS− enhanced), indicated by a significant main effect of time. We found no significant condition × session interaction in the fear recovery analysis for the contrast CS+ vs. CS−. However, the CS+/CS− difference was significant after reinstatement. This may be interpreted as weak evidence for differential return of fear. A review by Haaker et al. (2014) revealed heterogeneous reinstatement effects in humans. Some studies reported nondifferential (CS+ and CS− responses enhanced) or differential (only CS+ responses enhanced) return of fear while others failed to observe a reinstatement effect. According to this review, one may not be able to robustly expect reinstatement to provoke return of fear. Hence, there may be more suitable methods to investigate whether fear memory was reversed.

Several studies have reported discrepant drug effects on consolidation (Bach et al. 2018b) and reconsolidation (Soeter and Kindt 2010) in fear-potentiated startle and SCR. Although both measures are apparently influenced by amygdala-dependent threat learning, an additional impact of declarative memory may be different for the two measures (Ojala and Bach 2019) but this is not fully clear despite a body of literature. Elucidating the learning systems underlying different fear learning indices could thus be of utmost importance for developing targeted clinical applications.

The contrasting findings in the reminder/extinction literature initiated an intense debate on boundary conditions determining the efficacy of this approach. Reconsolidation is regarded as a fundamental property of memory but its induction seems to depend on specific requirements (Schwabe et al. 2014). Hence, certain reminder features have been highlighted to play an important role in the induction of reconsolidation and subsequent modification of the underlying memory trace, and to determine whether reminder leads to simple memory expression, reconsolidation or new learning (for review, see Fernández et al. 2016). Fernández et al. (2016) proposed that a prediction error generated by retrieval is essential to trigger reconsolidation and memory destabilization might be proportional to the generated prediction error. In contrast, Gershman et al. (2017) suggested that small prediction errors may induce modification of the original memory but large prediction errors, when retrieval conditions are significantly different to initial fear acquisition, rather induce new learning than memory update. Memory strength and age seem to be further boundary conditions for reconsolidation update but up to now it is unclear which factors are essential to trigger memory update.

In summary, we found no evidence that reminding a CS-US memory before extinction prevents reinstatement of PSR and SCR, i.e., the return of fear. We note that the reinstatement effect, although observed in the present study, may not be robust enough to reliably quantify the return of fear, and more effective procedures could improve experimental research in this area. Some degree of heterogeneity may be accounted for by the fact that different fear-conditioned measures index distinct components of the underlying learning process, and that CS-US contingency knowledge can impact on some of these measures as well. It appears that further experiments are needed to determine whether indeed fear memory can be modified by a behavioral intervention.

## Materials and Methods

### Power analysis

To determine required sample size, we conducted a power analysis (using G*power (Faul et al. 2007)) based on the effect magnitude stated in Schiller et al. (2010) and the measurement variability in a previous study in our group with the same setup (Korn et al. 2017), in which the effect size for a CS+/CS− difference in PSR was (Cohen's) *d* = 0.66. Notably, Schiller et al. (2010) did not provide an effect size for their main within-group finding such that the variability of the intervention could not be taken into account. We defined a fear memory reduction of at least 50% as relevant, in line with the about 60% reduction reported by Schiller et al. (2010). Under the best-case assumption that variance of the outcome measure is not affected by the intervention and that the intervention itself has no variability across participants, a sample size of *N* = 68 was required to achieve 85% power at an alpha rate of 0.05 to detect an at least 50% reduction in fear memory.

### Participants

We recruited 74 participants from the general population who reported to be healthy and had no history of psychiatric or neurological diseases. We excluded three participants who did not complete all 3 d, resulting in a sample size of 71 participants (38 females, aged 18–39 yr, 24.21 ± 0.46). The study was conducted in accordance with the Declaration of Helsinki and approved by the governmental research ethics committee (Kantonale Ethikkommission Zürich). All data are available on https://doi.org/10.5281/zenodo.3555306 (Zimmermann and Bach 2020).

In case of recording failures (e.g., electrode detachment) on one of the three study days, we excluded participants only for this particular day. In the PSR analysis, participants who had more than 50% trials with at least 50% missing data points over an experimental session were excluded from analysis of that experimental session. The numbers of participants included in the final analysis are listed in Table 4.

**Table 4.**
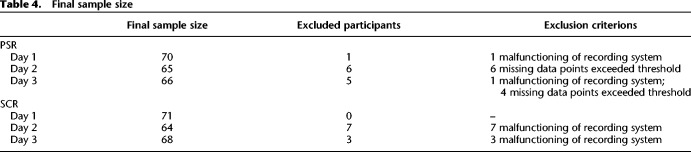
Final sample size

### Experimental design and procedure

**Figure 3.**
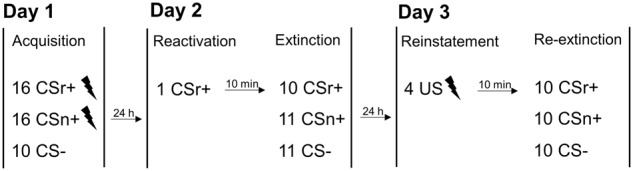
Experimental design: Participants underwent fear conditioning on day 1 including two CS+ conditions (CSr+/CSn+). On the subsequent day, only CSr+ was reminded before extinction and fear retention was tested on the following day in a reextinction session.

The experiment used a 3 conditions × 3 experimental sessions repeated-measures design to assess the return of fear, similar to Experiment 2 of Schiller et al. (2010). On day 1, participants underwent fear conditioning with two CS+ (CSr+/CSn+) and one CS−. CSr+ was reminded 10 min before the extinction session on day 2. On day 3, return of fear for both CS+ was assessed in a reextinction session after reinstatement. An overview on the experimental design used in our experiments is shown in Figure 3.

**Day 1:** Fear acquisition consisted of 10 CS− and 32 CS+ trials (16 CSr+/16 CSn+). Half of the CSr+/CSn+ trials were paired with a mild shock to the forearm (US). CS− stimuli were never followed by an US. All experiments were performed between 8 a.m. and 1 p.m.

**Day 2:** One day later, only CSr+ was reminded (single unreinforced trial) before extinction. A 10 min break separated the reminder trial from the extinction session, during which the participants stayed attached to the recording electrodes but were explicitly instructed that no shock would be delivered. During the break all participants watched a preselected TV show without audio but subtitles. The extinction session consisted of 11 CS−, 10 CSr+, and 11 CSn+ trials, all of which were nonreinforced.

**Day 3:** On the following day, participants received reinstatement, consisting of four unsignaled shocks (US alone). After a 10 min break, where participants watched a preselected TV show, we tested return of fear in a reextinction session. Ten unreinforced trials of each condition (CS−/CSr+/CSn+) were presented.

In the beginning of the experiment on day 1, participants were instructed that some stimuli may be paired with electric stimulation but were not informed about the contingencies. Participants had to press a specific key for the color of the stimulus on every trial to keep their attention. After stimulus offset, participants received feedback on the accuracy of their response. In a short training at the beginning of the experiment participants were instructed which key to press for the colors but were not told which condition was assigned to the color. The keys were counterbalanced across subjects. They were instructed that their response had no impact on the US.

During extinction and reextinction, the electrode for electric stimulation was attached to the participant's forearm to maintain participant's expectancy of a shock. At the end of each experimental session participants were tasked to rate shock expectancy for the different conditions.

### Stimuli and timings

We used colored triangles as CS+/CS− (yellow/magenta/cyan) on a gray background screen. The association of colors to conditions was randomized and balanced between subjects. During the intertrial interval, a gray screen with a fixation cross in the center was displayed. The colors of the CS, the background and the intertrial screen were adjusted to the same luminance (light emission per unit) to suppress changes in pupil size due to illuminance (perceived light emission from screen) variations during the experiment (RGB values stimuli: yellow (255, 176, 0); magenta (255, 124, 255); cyan (0, 255, 255); RGB values background and intertrial screen: 178.5, 178.5, 178.5). The CS presentation lasted 4 sec. In the reinforced CS+ trials the shock followed CS onset after 3.5 sec and coterminated with the CS. The intertrial interval was randomly determined as integer value between 7 and 11 sec (mean 9 sec). The trial order in each experiment and the intertrial interval were randomly generated. All experiments were programmed using MATLAB (Version R2018a, Math-Works) and Cogent 2000 toolbox (www.visilab.ucl.ac.uk).

The electrode (pin-cathode/ring-anode configuration) for electric stimulation was attached to participant's right forearm 10 cm from the distal wrist crease. Electric stimulation was delivered via a constant current stimulator (Digitimer DS7A, Digitimer Ltd). The US consisted of a 500-msec train of 250 square pulses with individual pulse width of 0.2 msec. US intensity was set individually for each participant to a certainly unpleasant but not painful level. For the calibration of the shock intensity, we increased the intensity gradually from a nonperceptible level and participants were asked to indicate the point when stimulation became clearly painful. This intensity was set as maximum for the second phase of calibration in which participants had to rate 14 stimuli, randomly generated in the range up to the defined maximum current. According to the ratings (0% = no shock perceived, up to 100% = painful shock) the definite intensity was determined as the rating at 85% (just below the reported pain threshold). Intensity calibration was only performed on the first day and the obtained intensity was kept constant over the following days (mean intensity ± SD: 3.58 ± 0.18 mA).

### Data recording

We recorded pupil diameter and gaze direction for both eyes with an EyeLink 1000 System (SR Research). The sampling rate was 500 Hz. To calibrate gaze direction we used the nine-point protocol implemented in the EyeLink 1000 software. The experiments occurred in a dark, soundproof chamber. Participants placed their head on a chin rest at a distance of 70 cm from the monitor (Dell P2012H, 20 inch set to an aspect ratio of 5:4, 60 Hz refresh rate).

Skin conductance electrodes were placed on the thenar/hypothenar of the left hand. We used 8 mm Ag/AgCl cup electrodes (EL258, Biopac Systems Inc.) filled with electrolyte gel (0.5% NaCl, GEL101, Biopac Systems Inc. (Hygge and Hugdahl 1985)). Skin conductance signal was amplified with a SCR coupler/amplifier (V71-23, Coulbourn Instruments). The output signal was digitized at a sampling rate of 1000 Hz using a DI-149 AD converter (Dataq Inc.) and recorded with Windaq (Dataq Inc.) software.

### Data processing

To process and analyze the psychophysiological data we used MATLAB (Version R2018a, Math-Works) and PsPM (Psychophysiological modelling, http://pspm.sourceforge.net, Version 4.0.2), a MATLAB toolbox for model-based analysis of psychophysiological data (Bach and Friston 2013; Bach et al. 2018a).

Pupil size data for which gaze direction was outside ±5° visual angle were treated as missing data points. The pupil with less missing data was used for subsequent analysis. Participants were excluded from analysis of an experimental session if more than 50% of the trials (CS onset + 7 sec) exceeded 50% missing data points. We *z*-scored the entire pupil data for the within experimental session analysis but not for multiple-day comparisons before downsampling the data to 250 Hz. To estimate the anticipatory pupil response, we used the single-trial general linear convolution model (GLM) implemented in PsPM developed by Korn et al. (2017).

We filtered SCR data (first order bidirectional band-pass Butterworth filter, 0.0159–5 Hz) and downsampled the data to 10 Hz as in Bach et al. (2010). To estimate the amplitudes of anticipatory SCR we used the dynamic causal model (DCM) implemented in PsPM (Bach et al. 2010; Staib et al. 2015). The DCM analysis provides trial by trial estimates of an anticipatory sudomotor burst, modeled as a Gaussian impulse (Bach et al. 2010). In line with intraneural recording results (Gerster et al. 2018), we fixed the sudomotor burst duration (SD = 0.3 sec) and constrained the (central) latency of the burst between 0 and 2.5 sec. The estimated response amplitudes used in subsequent statistical analysis (CS− and nonreinforced CS+ trials) were *z*-scored within participants per experimental session (Staib et al. 2015). For the analysis comparing responses across 2 d, we used nonnormalized DCM estimates.

### Statistical analysis

For statistical analysis we included only unreinforced trials. We applied a linear mixed effect model (LME) with fixed factors condition and time (trial number), an interaction term and a subject-specific intercept (random factor), using the R model formula:

amplitude∼1+condition×time,random=∼1|subj.

We performed an LME separately for each experimental session and the a priori contrasts CS+ (merging CSr+ and CSn+) vs. CS− and CSr+ vs. CSn+. Fear recovery was analyzed with a CS × session (extinction/reextinction) LME including the last three trials of extinction and the first three trials of reextinction after reinstatement for the same contrasts.

To allow direct comparison with the Schiller et al. (2010) study we used a two-way ANOVA with main effects condition (CS−, CSr+, CSn+) and time (first and second half of the reextinction session). Furthermore, we tested, for each condition, a fear recovery index as the difference between the last trial of extinction and the first trial of reextinction separately for each condition.

Statistical analysis was performed in RStudio (Version 2016; RStudio: Integrated Development for R., RStudio Inc., Boston, MA, USA). The linear mixed effect model (LME) was computed using nlme-package (*nlme: Linear and Nonlinear Mixed Effects Models*, R package version 3.1-131, https://CRAN.R-project.org/package=nlme). Fixed effects of the LME were extracted using the function anova() in RStudio. Bayesian information criterion (BIC) was extracted for maximum-likelihood fitted LMEs using the function BIC(). For the ANOVA we used the function aov() and for the paired *t*-tests the function t.test() implemented in RStudio.

## Supplementary Material

Supplemental Material
