# Supplementary material for: Impact of a reminder/extinction procedure on threat-conditioned pupil size and skin conductance responses
Source: Learn Mem. 2020 Apr;27(4):164–72. doi: 10.1101/lm.050211.119 (PMC7079572; doi:10.1101/lm.050211.119)
Supplement: Supplemental Material [file supp_27_4_164__index.html]

Impact of a reminder/extinction procedure on threat-conditioned pupil size and skin conductance responses — Supplemental Material 

# Impact of a reminder/extinction procedure on threat-conditioned pupil size and skin conductance responses

## Supplemental Material

- Supplemental\_Material.pdf
