# Supplementary material for: Impact of a reminder/extinction procedure on threat-conditioned pupil size and skin conductance responses
Source: Learn Mem. 2020 Apr;27(4):164–72. doi: 10.1101/lm.050211.119 (PMC7079572; doi:10.1101/lm.050211.119)
Supplement: Supplemental Material [file supp_27.4.164_Supplemental_Material.pdf]

Supplemental material for Zimmermann & Bach (2020), Impact of a reminder/extinction procedure on threat-conditioned pupil size and skin conductance responses, Learning & Memory

Supplementary analysis of fear acquisition

Fear acquisition on day 1 indicated near significant higher responses for CSn+ than CSr+ when analysing across the entire acquisition session. However, for our purposes we were mainly interested if both CS+ were ultimately learned to the same extent. Therefore, we performed an additional LME to verify if both CS+ conditions evoked equal responses during the second half of acquisition (see *Table S1* and *Table S2*). Indeed, CSr+ and CSn+ did not differ during the late phase of acquisition. The difference in learning when comparing all trials was strongly affected by the first acquisition trial. When only excluding the first trial per condition, fear acquisition showed similar responses for CSr+ and CSn+ (see *Table S1* and *Table S2*).

Table S1: Supplementary analysis of fear acquisition for PSR

	Contrast	Factor	F-value	df	p-value
**Day 1: Acquisition (without first trial per condition)**	CS+ vs. CS-	Condition	15.19	1, 1318	**<0.001**
		Time (linear)	241.75	1, 1318	**<0.001**
		Cond. × time	5.06	1, 1318	**0.025**
	CSr+ vs. CSn+	Condition	2.70	1, 773	0.10
		Time (linear)	116.80	1, 773	**<0.001**
		Cond. × time	1.24	1, 773	0.27
**Day 1: Acquisition (second half)**	CS+ vs. CS-	Condition	20.24	1, 697	**<0.001**
		Time (linear)	8.64	1, 697	**0.003**
		Cond. × time	2.37	1, 697	0.12
	CSr+ vs. CSn+	Condition	0.03	1, 400	0.87
		Time (linear)	2.50	1, 400	0.11
		Cond. × time	0.14	1, 400	0.71

df: degrees of freedoms
Significant results are written in bold

Table S2: Supplementary analysis of fear acquisition for SCR

	Contrast	Factor	F-value	df	p-value
**Day 1: Acquisition (without first trial per condition)**	CS+ vs. CS-	Condition	12.85	1, 1559	**0.003**
		Time (linear)	26.08	1, 1559	**<0.001**
		Cond. × time	0.04	1, 1559	0.85
	CSr+ vs. CSn+	Condition	2.61	1, 920	0.11
		Time (linear)	14.04	1, 920	**<0.001**
		Cond. × time	0.82	1, 920	0.37
**Day 1: Acquisition (second half)**	CS+ vs. CS-	Condition	7.68	1, 849	**0.006**
		Time (linear)	5.32	1, 849	**0.021**
		Cond. × time	0.01	1, 849	0.99
	CSr+ vs. CSn+	Condition	0.01	1, 494	0.91
		Time (linear)	3.42	1, 494	0.065
		Cond. × time	6.69	1, 494	**0.010**

df: degrees of freedoms
Significant results are written in bold

Results of complete re-extinction session

Analysis of the complete re-extinction session revealed significant difference for CS+ vs. CS- (main effect condition) in PSR and SCR (see *Table S3* and *Table S4*). The reminded (CSr+) and the non-reminded (CSn+) stimulus evoked equal responses. These results are consistent with the analysis of the first three trials of the re-extinction session.

Table S3: Results of complete re-extinction session for PSR

	Contrast	Factor	F-value	df	p-value
**Day 3: Re-extinction**	CS+ vs. CS-	Condition	19.38	1, 1702	**<0.001**
		Time (linear)	355.96	1, 1702	**<0.001**
		Cond. × time	1.21	1, 1702	0.27
	CSr+ vs. CSn+	Condition	0.78	1, 1116	0.38
		Time (linear)	245.77	1, 1116	**<0.001**
		Cond. × time	0.77	1, 1116	0.38

df: degrees of freedoms
Significant results are written in bold

Table S4: Results of complete re-extinction session for SCR

	Contrast	Factor	F-value	df	p-value
**Day 3: Re-extinction**	CS+ vs. CS-	Condition	6.84	1, 1969	**0.009**
		Time (linear)	66.23	1, 1969	**<0.001**
		Cond. × time	4.53	1, 1969	**0.034**
	CSr+ vs. CSn+	Condition	0.01	1, 1289	0.96
		Time (linear)	62.03	1, 1289	**<0.001**
		Cond. × time	0.27	1, 1289	0.60

df: degrees of freedoms
Significant results are written in bold

Results after excluding subjects according to the criteria used in Schiller et al. (2010)

To make our study directly comparable to the study of Schiller et al. (2010), we applied the exclusion criteria provided in the addendum to the original study (Schiller et al. 2018). The complete exclusion criteria are listed at the end of this document.

For the analysis of PSR, we excluded 9 participants due to acquisition failure and 2 participants who did not show adequate extinction. We included only participants with valid data on both days, resulting in 53 participants remaining out of the original sample of 71 participants. For the analysis of re-extinction and fear recovery, three participants were additionally excluded due to invalid recordings on day 3.

In PSR, we observed successful fear learning indicated by a main effect of condition, time and a condition × time interaction. CSr+ and CSn+ were equally learned (see *Table S5*). In extinction, we observed a significant main effect of condition for CS+/CS- and CSr+/CSn+. However, both CS+ stimuli evoked equal responses in the last trial of extinction (CSr+ vs. CSn+: $t(52) = 1.11$, $p = 0.27$) and both CS+ were similar to CS- (CSr+ vs CS-: $t(52) = 1.38$, $p = 0.17$; CSn+ vs CS-: $t(52) = 0.06$, $p = 0.96$). The analysis of the first three trials of re-extinction and fear recovery revealed no difference between the reminded and the non-reminded stimuli (see *Table S5*). Comparing model evidence for the full model with a reduced model that did not separate CSr+ and CSn+, evidence was in favour of the reduced model for the first three trials after reinstatement (LBF = 4.20) and fear recovery (LBF = 5.13). In an additional analysis equivalent to Schiller et al. (2010), we tested the last trial of extinction to the first trial of re-

extinction for all three conditions. We observed significantly stronger responses after reinstatement for all three conditions (CS-: $t(49) = 6.00$, $p < 0.001$; CSr+: $t(49) = 6.21$, $p < 0.001$; CSn+: $t(49) = 6.20$, $p < 0.001$). When testing CSr+ against CSn+ in the difference between the last trial of extinction and the first trial after reinstatement, we detected no difference in the recovery of fear ($t(49) = 0.20$, $p = 0.84$).

According to the SCR responses, we excluded 47 participants due to acquisition failure and 2 participants because they did not display sufficient extinction. Finally, we included 22 participants out of the original sample.

SCR were higher for CS+ than CS- (main effect condition) and both CS+ conditions were equally learned (see *Table S6*). In extinction, we found no main effect of condition for the contrast CS+/CS-and for CSr+/CSn+. During the last trial of extinction both CS+ evoked equal responses (CSr+ vs. CSn+: $t(21) = 0.03$, $p = 0.98$) and both were similar to CS- (CSr+ vs CS-: $t(21) = 1.66$, $p = 0.11$; CSn+ vs CS-: $t(21) = 0.93$, $p = 0.36$). In contrast to Schiller et al. (2010), the reminded and the non-reminded stimuli evoked equal responses when analysing the first three trials of re-extinction but also in fear recovery (see *Table S6*). Model evidence favoured a reduced model for the first three trials of re-extinction (LBF = 4.27) and fear recovery (LBF = 4.57). Interestingly, in the analysis Schiller et al. (2010) performed in their original study, we found a significant return of fear only for the non-reminded CSn+ stimuli (CSn+: $t(21) = 2.42$, $p = 0.025$), while the reminded stimuli evoked only near-significant higher responses after reinstatement (CSr+: $t(21) = 1.89$, $p = 0.073$). CS- responses were similar before and after reinstatement (CS-: $t(21) = 1.52$, $p = 0.14$). However, comparing CSr+ against CSn+ in the difference from the last trial of extinction to the first trial after reinstatement showed equal return of fear for both conditions and not even a trend in this direction (CSr+ vs. CSn+: $t(21) = 0.25$, $p = 0.80$). Notably, this direct comparison between the reminded and the non-reminded stimuli was lacking in the analysis of Schiller et al. (2010).

Table S5: Results after applying Schiller exclusion criteria for PSR

	Contrast	Factor	F-value	df	p-value
**Day 1: Acquisition**	CS+ vs. CS-	Condition	11.75	1, 1128	**<0.001**
		Time (linear)	362.38	1, 1128	**<0.001**
		Cond. × time	4.90	1, 1128	**0.027**
	CSr+ vs. CSn+	Condition	1.66	1, 675	0.20
		Time (linear)	187.55	1, 675	**<0.001**
		Cond. × time	0.56	1, 675	0.46
**Day 2: Extinction**	CS+ vs. CS-	Condition	14.52	1, 1438	**<0.001**
		Time (linear)	279.44	1, 1438	**<0.001**
		Cond. × time	2.34	1, 1438	0.13
	CSr+ vs. CSn+	Condition	4.91	1, 934	**0.027**
		Time (linear)	208.52	1, 934	**<0.001**
		Cond. × time	1.45	1, 934	0.23
**Day 3: Re-extinction (first 3 trial)**	CS+ vs. CS-	Condition	17.92	1, 350	**<0.001**
		Time (linear)	138.56	1, 350	**<0.001**
		Cond. × time	5.26	1, 350	**0.022**
	CSr+ vs. CSn+	Condition	2.19	1, 218	0.14
		Time (linear)	113.71	1, 218	**<0.001**
		Cond. × time	1.75	1, 218	0.19
**Fear recovery**	CS+ vs. CS-	Condition	11.78	1, 759	**<0.001**
		Session (extinction/re-extinction)	127.38	1, 759	**<0.001**
		Cond. × session	0.80	1, 759	0.37
	CSr+ vs. CSn+	Condition	1.67	1, 493	0.20
		Session (extinction/re-extinction)	92.31	1, 493	**<0.001**
		Cond. × session	0.15	1, 493	0.70

Significant results are written in bold
df: degrees of freedom
Fear recovery: including last 3 trials of extinction on day 2 vs. first 3 trials of re-extinction on day 3

Table S6: Results after applying Schiller exclusion criteria for SCR

	Contrast	Factor	F-value	df	p-value
**Day 1: Acquisition**	CS+ vs. CS-	Condition	4.88	1, 547	**0.028**
		Time (linear)	12.73	1, 547	**<0.001**
		Cond. × time	0.68	1, 547	0.41
	CSr+ vs. CSn+	Condition	0.68	1, 327	0.41
		Time (linear)	11.18	1, 327	**<0.001**
		Cond. × time	0.58	1, 327	0.45
**Day 2: Extinction**	CS+ vs. CS-	Condition	0.18	1, 679	0.67
		Time (linear)	4.97	1, 679	**0.026**
		Cond. × time	0.17	1, 679	0.68
	CSr+ vs. CSn+	Condition	0.29	1, 437	0.59
		Time (linear)	2.40	1, 437	0.12
		Cond. × time	1.35	1, 437	0.25
**Day 3: Re-extinction (first 3 trial)**	CS+ vs. CS-	Condition	3.54	1, 173	0.061
		Time (linear)	0.44	1, 173	0.50
		Cond. × time	0.92	1, 173	0.34
	CSr+ vs. CSn+	Condition	0.85	1, 107	0.36
		Time (linear)	1.01	1, 107	0.32
		Cond. × time	0.38	1, 107	0.54
**Fear recovery**	CS+ vs. CS-	Condition	2.17	1, 371	0.14
		Session (extinction/re-extinction)	41.81	1, 371	**<0.001**
		Cond. × session	0.69	1, 371	0.41
	CSr+ vs. CSn+	Condition	1.98	1, 239	0.16
		Session (extinction/re-extinction)	29.92	1, 239	**<0.001**
		Cond. × session	0.02	1, 239	0.88

Significant results are written in bold
df: degrees of freedom
Fear recovery: including last 3 trials of extinction on day 2 vs. first 3 trials of re-extinction on day 3

The exclusion criteria used in Schiller et al. (2010)

"Specifically, 'successful acquisition and extinction' for the participants (…) can be translated to a chain of logical "IF" statements:

- If during acquisition the differential CS response (CS+ minus CS-) was below an individually standardized cut-off (value of 0.1 divided by the mean US response) on: (1) the first half of acquisition, (2) the second half acquisition, (3) last trial of acquisition, and (4) the increase from the first to last trial

of acquisition. When *all* of these criteria were met then acquisition was deemed as failed, otherwise the participant was included.

- If during extinction the differential CS response (CS+ minus CS-) was above an individually standardized cut-off on: 1) the second half of extinction, 2) the last trial of extinction, and 3) below cutoff in the decrease from the first to the last trials or halves of extinction. When *all* of these criteria were met then extinction was deemed as failed, otherwise the participant was included." (Schiller et al. 2018)

References

Schiller D, Monfils M-H, Raio CM, Johnson DC, Ledoux JE, Phelps E a. 2010. Preventing the return of fear in humans using reconsolidation update mechanisms. *Nature* **463**: 49–53. http://dx.doi.org/10.1038/nature08637.

Schiller D, Monfils MH, Raio CM, Johnson DC, LeDoux JE, Phelps EA. 2018. Erratum: Addendum: Preventing the return of fear in humans using reconsolidation update mechanisms (Nature (2010) 463 7277 (49-53)). *Nature* **562**: E21. http://dx.doi.org/10.1038/s41586-018-0405-7.
